# Development and characterization of chromosome segment substitution lines derived from *Oryza rufipogon* in the genetic background of *O. sativa spp. indica* cultivar 9311

**DOI:** 10.1186/s12864-016-2987-5

**Published:** 2016-08-09

**Authors:** Weihua Qiao, Lan Qi, Zhijun Cheng, Long Su, Jing Li, Yan Sun, Junfang Ren, Xiaoming Zheng, Qingwen Yang

**Affiliations:** 1 Institute of Crop Science, Chinese Academy of Agricultural Sciences, 12 Zhongguancun South Street, Beijing, Haidian 100081 China; 2 Institute of Cereal Crop Science, Hainan Academy of Agricultural Sciences, 14 Xingdan Road, Haikou, Hainan 571100 China; 3 Institute of Tropical Horticulture, Hainan Academy of Agricultural Sciences, 14 Xingdan Road, Haikou, Hainan 571100 China

**Keywords:** Chromosome segment substitution lines, *Oryza rufipogon*, Quantitative trait locus

## Abstract

**Background:**

Wild rice (*Oryza rufipogon*) constitutes a primary gene source for rice breed improvement. Chromosome segment substitution line (CSSL) for *O. rufipogon* is a powerful tool for fine mapping of quantitative traits, new gene discovery, and marker-assisted breeding. Thus, they provide a basis for a wide range of genomic and genetic studies.

**Results:**

In this study, a set of 198 CSSLs were developed from a cross between recurrent parent *indica* var. 9311 and an *O. rufipogon* donor parent; these were then genotyped using 313 polymorphic SSR markers evenly distributed across the 12 rice chromosomes. On average, each CSSL carried 2.16 introgressed segments, and the genetic distance of each segment was about 6 cM. The segments collectively covered 84.9 % of the wild rice genome. Based on these CSSLs, 25 QTLs involved in 10 agronomic traits were identified. Seven CSSLs were subjected to a whole-genome single nucleotide polymorphism chip assay and two QTLs, *qSH4-1* and *qDTH10-1*, detected. In addition, a new QTL associated with the heading date was detected in a 78-Kb region on chromosome 10, thus proving the ability of these CSSLs to identify new QTLs and genes.

**Conclusions:**

The newly developed CSSL population proved a useful tool for both gene identification and whole-genome research of wild rice. These CSSL materials will provide a foundation for rice variety improvement.

**Electronic supplementary material:**

The online version of this article (doi:10.1186/s12864-016-2987-5) contains supplementary material, which is available to authorized users.

## Background

A problem facing traditional rice breeding is that of yield plateaus, these are caused by the narrow genetic basis of parental materials [1]. The transfer of genes controlling desirable traits from wild relatives to cultivated rice is an important strategy in rice breeding. The O*ryza* family, which includes cultivated rice and wild species, contains highly diverse geographical, morphological, and physiological characteristics [2]. Wild *Oryza* species with 2n = 24 or 48 chromosomes and genome constitutions AA, BB, CC, BBCC, CCDD, EE, FF, GG, or HHJJ are important reservoirs of genes with potential for use in rice breeding [3].

Common wild rice (*Oryza rufipogon* Griff.) has a similar AA genome to cultivated rice, and is considered the direct ancestor of cultivated rice (*Oryza sativa* L.) [4–6]. During the course of domestication to cultivated rice, many desirable traits, such as resistance to diseases and pests, and adaptation to unfavorable environments were lost, profoundly decreasing genetic diversity [7–9]. Many genes controlling important agronomic traits in rice domestication, such as days to heading, seed shattering, and seed dormancy, have been found in wild rice relatives [10–12]. Although the overall economical characters of wild rice are inferior to those of cultivated rice, modern molecular biology studies have revealed potential genes hidden in wild rice are essential for yield-related trait improvement [13, 14]. Therefore, it is important to discover these useful genes and to apply their use in rice breeding programs.

Many important traits in rice, including heading date, culm length, eating quality, and yield are controlled by quantitative trait loci (QTL) and show continuous phenotypic variation in progenies. Construction and use of a suitable genetic population is pivotal for fine mapping of QTLs and map-based cloning of target genes. Temporary mapping populations such as F_2_ or BC_1_ [15–17] and permanent primary mapping populations including doubled-haploid and recombinant inbred lines have been developed for genetic analysis of complex traits [18, 19]. However, these mapping populations cannot be used to estimate individual QTLs precisely owing to genetic background noise [20, 21]. Thus, they are not adequate for fine mapping and characterization of target QTLs and further analyses [22]. Furthermore, phenotypic effects of QTLs are always influenced by genetic backgrounds and environmental factors. Therefore, the development of advanced mapping populations such as introgression lines (ILs) and chromosome segment substitution lines (CSSLs) to analyze QTLs has received great attention. A CSSL population is generally developed through advanced backcrossing, selfing, and marker-assisted selection (MAS). In CSSL populations, each line carries a single or a few chromosomal segments from the donor parent in the genetic background of the recurrent parent. An ideal CSSL population is composed of lines carrying only one different chromosome segment from the donor parent, but with the whole population carrying the entire genome of the donor.

CSSLs from interspecific hybridization represent a powerful and useful genetic resource for genome research, especially QTL mapping and gene cloning, and for pyramiding target segments and breeding [23]. The first complete set of substitution lines were constructed in tomato by Eshed and Zamir [24, 25]. These consisted of near isogenic lines (NILs) carrying single *Lycopersicon pennellii* chromosomal segments in an otherwise homogeneous *L. esculentum* background; these represented the entire genome of wild tomato [24, 25]. At least 20 sets of ILs and CSSLs have been constructed in rice [26–32], and many agronomic QTLs have been identified and some cloned using map-based methods [30–36]. Tian, Tan, and their co-workers constructed two series of ILs for Yuanjiang and Dongxiang common wild rice in China, respectively [13, 37]. Hirabayashi *et al*. developed ILs from *O. rufipogon* and *Oryza glumaepatula* in a *japonica* cultivated rice background [38]. A difficulty with IL populations is that they do not cover the entire wild rice genome, making precise QTL mapping difficult. Recently, Furuta *et al.* developed 33 CSSLs of *O. rufipogon* in an elite *japonica* cultivar Koshihikari background using 149 single nucleotide polymorphism (SNP) markers [39]. However, this small population was not large enough for a better understanding of the wild rice genome, with long and redundant introgressive segments blocking fine mapping of new genes. Moreover, there are few CSSLs reported for wild rice from the low-latitude areas of China that use the sequenced *indica* cultivar background. Thus, to identify and employ desirable genes, and to have a better understanding of the genetic diversity of wild rice, it is necessary to construct new *O. rufipogon–O. sativa* CSSLs.

In this study, a broad population of 198 CSSLs was constructed from backcross progenies derived from a cross between the commercial indica cultivar 9311 as the recurrent parent and the wild rice CWR276 as the donor parent. The CSSL population was genotyped using 313 polymorphic simple sequence repeat (SSR) markers distributed evenly across the 12 rice chromosomes. The CSSL population covered 84.9 % of the wild rice genome with an average substituted segment length of 6 cM. Based on evaluation of the phenotypic variation of quantitative trait and identification of QTLs, seven CSSLs were selected for whole-genome SNP chip assays. Finally, a new QTL associated with the heading date was identified. We demonstrated this CSSL population as a useful tool not only for fine mapping of genes but also for wild rice genomic research. In addition, these CSSL materials provide a foundation for developing future rice cultivars for breeding programs.

## Results

### Identification of SSR markers for MAS

**Fig. 1:**
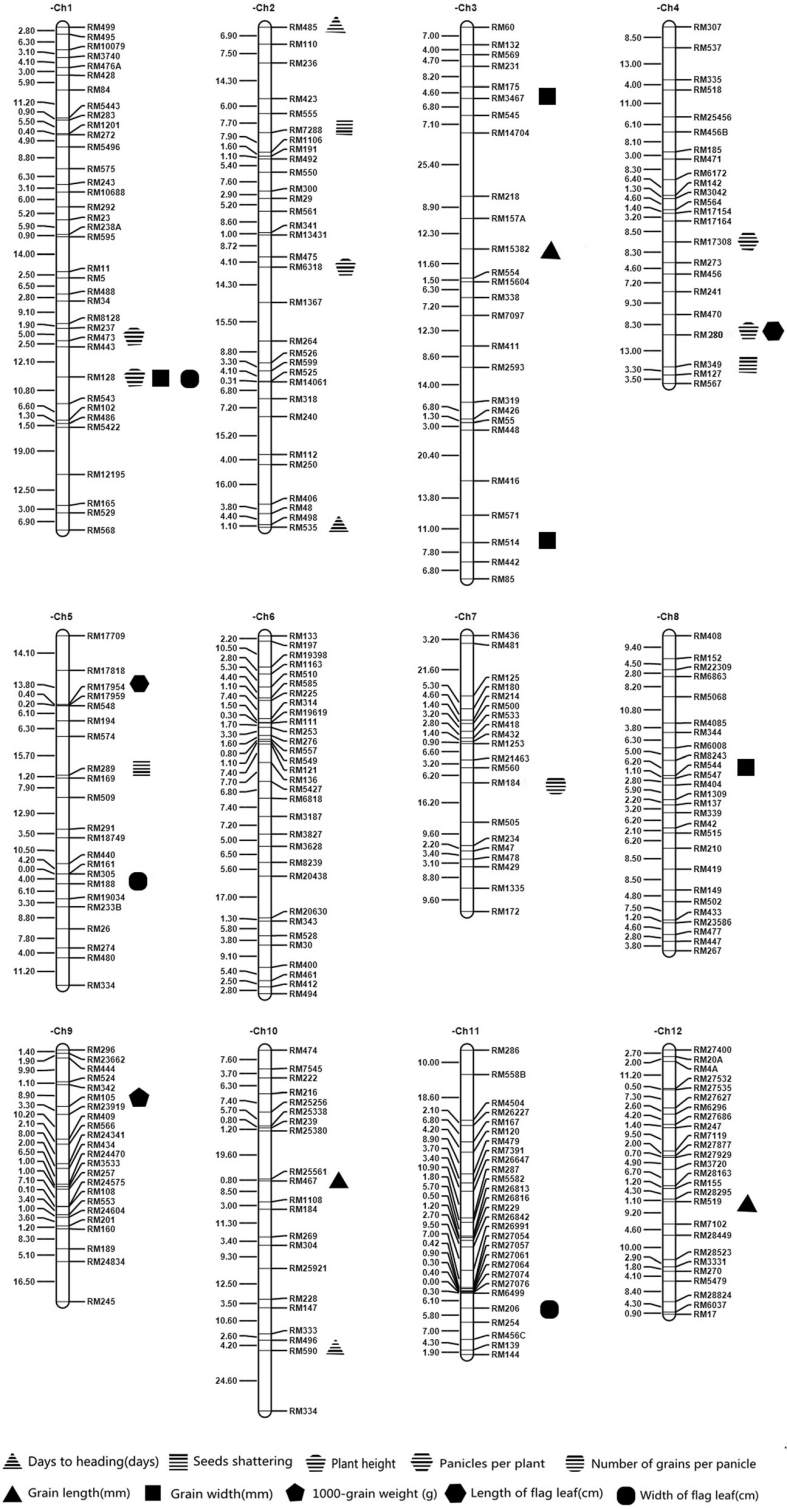
Genetic location of 313 polymorphic markers and distribution of 25 QTLs for 10 agronomic traits. Molecular markers are shown to the right of chromosomes, and genetic locations (cM) of each marker shown to the left of chromosomes. The regions of 25 detected QTLs are shown using a different shape

In all, 780 SSR markers distributed throughout the 12 rice chromosomes were used to detect polymorphisms between 9311 and CWR276, among which 369 (52.7 %) were polymorphic between these two parents. Finally, 313 polymorphic SSR markers were selected for analysis of the CSSL genotypes. Information regarding the genetic distance of these markers was downloaded from Gramene [40]. Sequence information of markers used in this study was showed in S-Table 1. The average distance between two adjacent markers on the rice linkage map was 5.7 cM, and ranged from 0.1 cM to 25 cM (Table 1; Fig. 1). The polymorphic markers were further used for MAS in the process of developing CSSLs and genotyping of the CSSL population.

**Table 1:** Distribution of polymorphic markers on the 12 chromosomes

Chromosome	Chromosome length (cM)	Average distance between 2 markers (cM)	No. of markers
Chr.1	202.3	5.62	36
Chr.2	201.3	6.49	31
Chr.3	221.4	8.51	26
Chr.4	144.9	6.30	23
Chr.5	142.0	6.45	22
Chr.6	145.3	4.68	31
Chr.7	113.3	5.66	20
Chr.8	128.4	4.94	26
Chr.9	103.6	4.50	23
Chr.10	146.6	6.98	21
Chr.11	124.4	4.44	28
Chr.12	108.5	4.17	26
Total	1782	5.69	313

### CSSL development

**Fig. 2:**
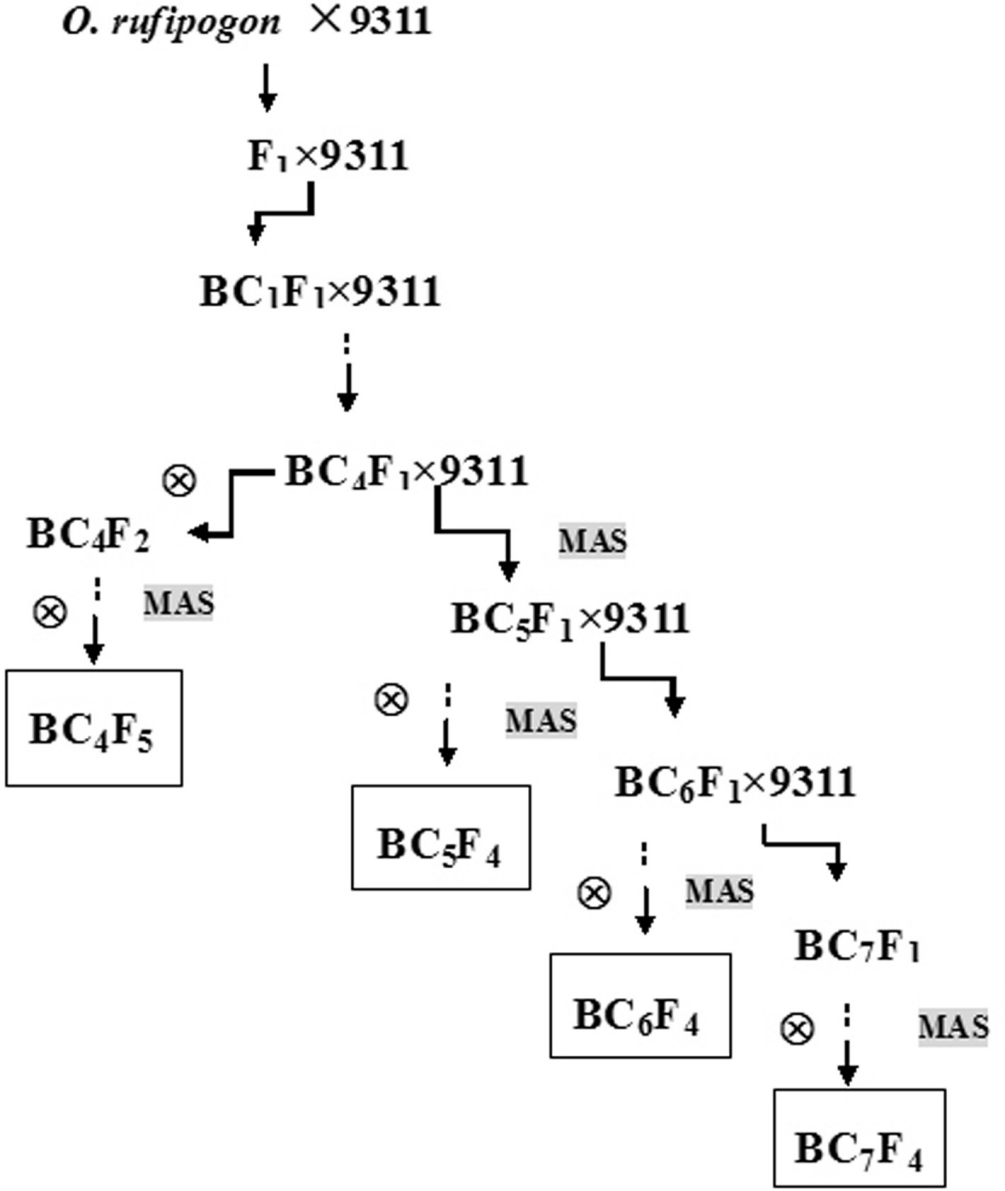
Schematic of the development of CSSLs carrying *O. rufipogon* (CWR276) chromosome segments in an *O. sativa inidca* 9311 genetic background

The procedure used for CSSL development is shown schematically in Fig. 2. Following the initial cross between the 9311 ‘female’ and the CWR274 ‘male’, var. 9311 was used as the recurrent parent to backcross the hybrid three times, obtaining BC_3_F_1_. Over 1,000 BC_3_F_1_ plants were investigated using 230 SSR markers distributed across the 12 chromosomes, and 236 plants were selected for further backcrossing. In the BC_4_F_1_ generation, 376 individuals were subjected to a whole-genome survey using 313 SSR markers. Fifty-eight plants with less than three substituted segments from wild rice were selected and successively self-crossed to produce CSSLs. Similarly, 65, 43, and 32 CSSLs from BC_5_F_1,_ BC_6_F_1_, and BC_7_F_1_, were obtained, respectively. Thus, a total set of 198 CSSLs lines was developed.

### Distribution, number, and length of substituted chromosome segments in CSSLs

**Fig. 3:**
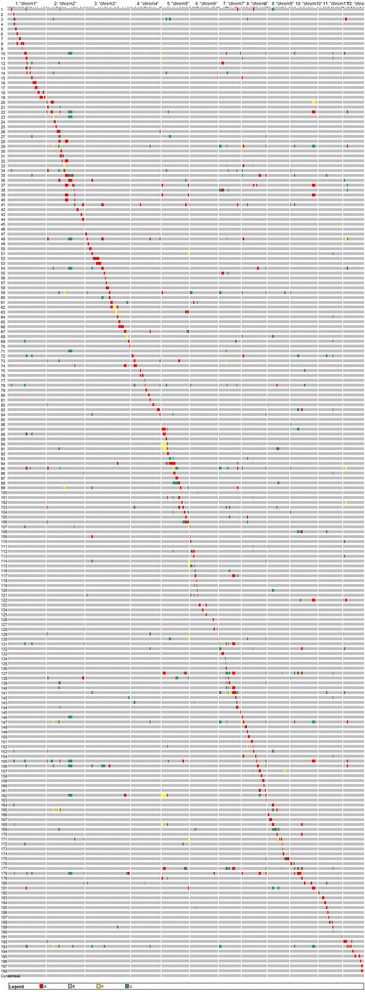
Graphic of genotypes of the 198 CSSLs. Regions with a gray background represent homozygous segments from 9311; red regions indicate homozygous segments from CWR276; yellow regions indicate heterozygous segments of the two parents. The green regions indicate a non-parent allele. The horizontal axis indicates one CSSL, and the vertical axis indicates one substituted segment of wild rice

The 198 CSSLs carried 412 homozygous introgressed chromosome segments and 72 heterozygous segments; on average each CSSL contained 2.16 wild rice segments (Table 2, Fig. 3). The CSSLs accumulatively covered 84.9 % (1531 cM) of the wild rice genome marker base. There was an uneven distribution among the 12 chromosomes, with most introgressed segments (77) found on chromosome 2, and chromosome 11 having the least (19). Seventy-two CSSLs carried single introgressed segments, these involved 37 heterozygous segments and 35 homozygous ones; these were considered NILs of the recurrent parent. However, the transmission and recombination of *O. rufipogon* substituted segments varied for each individual chromosome, with coverage for chromosome 7 being only 61.6 %, whereas chromosomes 2, 3, 5, and 10 had full coverage.

**Table 2:** Substitution of *O. rufipogon* segments in CSSLs and cumulative proportion of donor genome represented by homozygous and heterozygous segments

Chromosome	Homozygous segments		Heterozygous segments		Total segment Length (cM)	Effective coverage length (cM)	Genome coverage (%) (Het + Homo)
	Number of segments	Average length (cM)	Number of segments	Average length (cM)			
1	38	6.10	4	5.90	255.1	180.0	89.0
2	64	7.55	13	5.98	483.4	201.3	100
3	37	9.10	4	8.12	369.4	221.4	100
4	23	5.20	4	3.70	134.4	108.5	74.9
5	46	7.02	15	6.90	406.6	142.0	100
6	31	3.37	3	6.86	125.1	90.5	62.3
7	39	4.77	7	4.45	217.5	69.8	61.6
8	38	5.92	8	6.57	277.5	105.8	82.4
9	23	5.27	6	6.08	157.9	86.1	83.1
10	32	9.13	5	7.32	328.8	146.6	100
11	19	4.27	0	0	86.8	82.3	66.2
12	22	4.73	3	7.3	126.0	96.8	89.2
Average	2.16	6.03	0.38	5.76	(Total) 2968.5	(Total) 1531.1	84.9

Maximum percentage of genome coverage was based on the proportion of each chromosome’s genetic length being represented by at least one CSSL

**Fig. 4:**
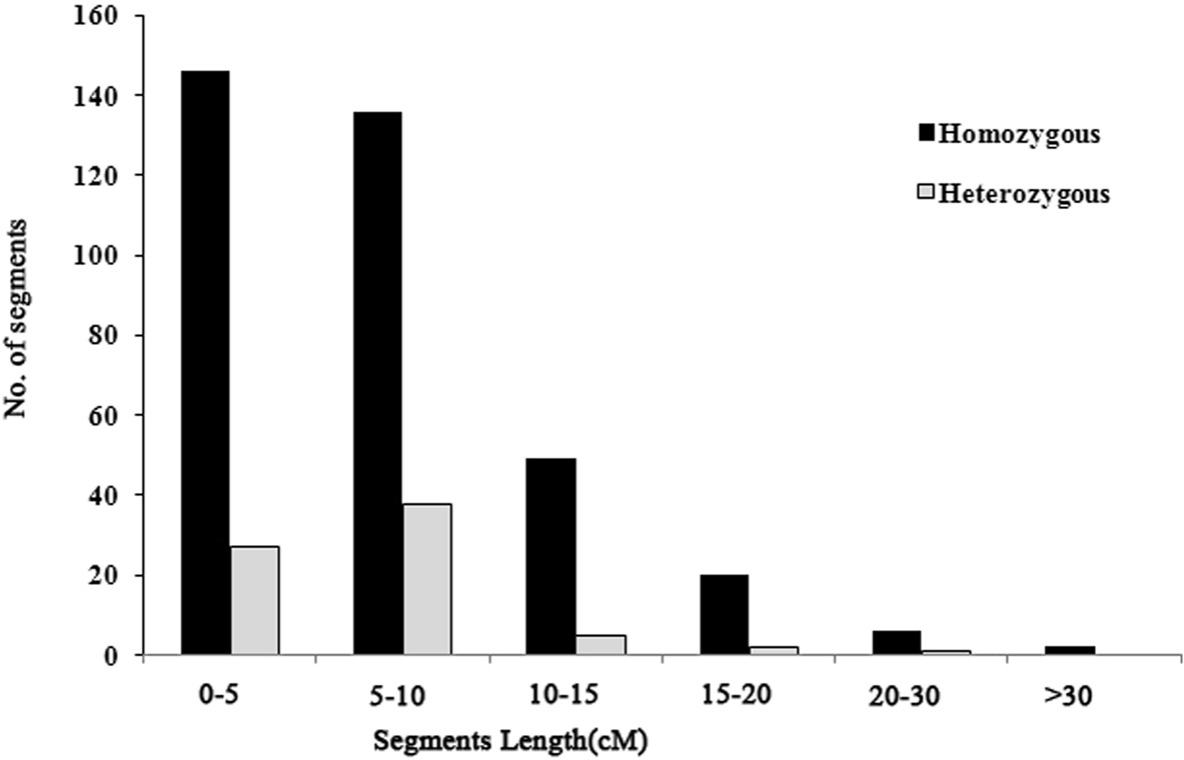
Frequency of substituted homozygous and heterozygous chromosome segments according to genetic length

Among the 198 CSSLs, sizes of the 484 substituted segments ranged from 0.7 cM (on Chr. 9 of CSSL157) to 34.4 cM (on Chr. 5 of CSSL 91), with an average of 6.03 cM. Forty percent of substituted segments were smaller than 5 cM, 40 % of substituted segments were from 5 to 10 cM, 18 % ranged from 10 to 20 cM, and 2 % of segments were over 20 cM (Fig. 4).

### Evaluation of phenotypic variation of quantitative traits in the CSSLs

Morphometric measurements of agronomic traits in the 198 CSSLs are presented in Table 3. The values of the 10 agronomic traits showed a large range of variation. All of the traits were observed to have phenotypic transgressive variation. Among the 10 investigated traits, seed shattering and number of grains per panicle were the most represented in the CSSLs, whereas days to heading was the least represented trait. The correlations among 10 agronomic traits were shown in Additional file 1: Table S2. In most cases, the correlation coefficients are significant or highly significant. Additionally, some domestication-related traits, such as lazy growth habit, red and long awn, spread panicle, and black hull were observed in the CSSLs (data not shown).

**Table 3:** Statistics for ten agronomic traits of 9311 and the 198 CSSLs population, observed at the Nanjing and Sanya locations

	9311	CSSLs (Nanjing)			9311	CSSLs (Sanya)		
Traits		Mean ± SD	CV%	Range		Mean ± SD	CV%	Range
Days to heading(days)	102	94.7 ± 4.26	3.70	75-123	120	120 ± 3.55	2.96	101-129
Seeds shattering	5	4.75 ± 1.9	40	1-9	5	4.75 ± 1.9	40	1-9
Plant height(cm)	124.4	106.55 ± 22.46	21.08	78.4-198.7	98.2	104.08 ± 19.73	18.95	73-181
Number of panicles per plant	6.6	10.41 ± 2.01	19.31	6.6-17.8	7.9	8.69 ± 1.46	16.74	5.4-14.6
Number of grains per panicle	158.2	151.7 ± 36.42	24.30	72.4-248.8	173.7	145.1 ± 37.61	25.90	73.8-248.7
Grain length(mm)	10.09	10.03 ± 0.63	6.27	8.44-11.78	9.22	9.25 ± 0.4	4.29	7.40-9.62
Grain width(mm)	2.84	2.8 ± 0.1	3.65	2.36-3.13	2.81	2.81 ± 0.12	4.40	2.09-3.32
1000-grain weight (g)	30.86	29.56 ± 2.7	9.13	21.73-36.52	31.93	29.88 ± 2.64	8.83	18.47-36.38
Length of flag leaf(cm)	33.1	26.78 ± 3.57	13.34	18.2-38.8	23.0	25.11 ± 4.14	16.74	15.7-45.1
Width of flag leaf(cm)	2.5	2.02 ± 0.21	10.16	1.4-2.7	1.7	1.69 ± 0.14	8.25	1.3-2.1

### QTL analysis

QTLs analysis for the 10 agronomic traits was carried out separately at both Nanjing and Sanya sites using IciMapping software [41]. Forty-five QTLs were detected in the CSSLs (Additional file 2: Table S3), with 25 significant QTLs identified at both sites (Table 4, Fig. 1).

**Table 4:** QTLs for 10 agronomic traits detected in the 198 CSSLs at both sites

Trait	QTL	Marker	Chromosome	PVE (%)	Add
Days to heading	*qDTH2-1*	RM485	2	17.3	20.6
	*qDTH2-2*	RM535	2	7.89	19.0
	*qDTH10-1*	RM590	10	15.3	21.2
Seed shattering	*qSH2-1*	RM7288	2	6.18	1.16
	*qSH4-1*	RM349	4	8.55	1.66
	*qSH5-1*	RM289	5	5.04	-0.76
Plant height	*qPH1-1*	RM128	1	7.01	15.9
	*qPH1-2*	RM473	1	9.05	25.3
	*qPH2-1*	RM6318	2	5.20	-17.6
	*qPH4-1*	RM280	4	8.43	34.5
Panicles per plant	*qPPP4-1*	RM17308	4	5.66	0.89
Number of grains per panicle	*qGPP7-1*	RM184	7	3.54	-50.2
Grain length	*qGL3-1*	RM15382	3	15.0	-0.45
	*qGL10-1*	RM467	10	6.07	0.11
	*qGL12-1*	RM519	12	15.3	0.27
Grain width	*qGW3-2*	RM514	3	11.2	-0.15
	*qGW8-1*	RM544	8	6.08	-0.06
	*qGW1-1*	RM128	1	11.3	-0.10
	*qGW3-1*	RM3467	3	19.0	0.10
1000-grain weight	*qTGW9-1*	RM105	9	8.07	-1.24
	*qTGW5-1*	RM188	5	11.9	-1.50
Length of flag leaf	*qLFL4-1*	RM280	4	18.6	10.6
	*qLFL5-1*	RM17954	5	4.50	2.62
Width of flag leaf	*qWFL11-1*	RM206	11	8.58	-0.09
	*qWFL1-1*	RM128	1	6.48	-0.10

The value of PVE (Phenotypic variation explained by the QTL) and Add (Estimated additive effect of the QTL) were from Nanjing site

### Days to heading

Three QTLs, located near RM485 and RM535 on Chr.2, and RM590 on Chr.10 were associated with days to heading. These loci showed an increasing effect on days to heading. Phenotypic variation explained by these three QTLs ranged from 7.89 to 17.3 %.

### Seed shattering

Three QTLs associated with seed shattering were detected at both sites. Two QTLs associated with increasing seed shattering were located near markers RM7288 on Chr.2 and RM349 on Chr.4. A QTL near RM289 on Chr.5 showed a decreasing effect on seed shattering. The phenotypic variation explained by these three QTLs ranged from 5.04 to 8.55 %.

### Plant height

Four QTLs associated with plant height were detected at both sites. The directions of their effects at both sites were the same. Three QTLs derived from the wild rice were located near markers RM128 and RM473 on Chr.1 and RM280 on Chr.3; these showed an increasing effect on plant height. A further QTL, near RM6318 on Chr.2, had a decreasing effect on plant height. The phenotypic variation explained by these four QTLs ranged from 5.20 to 9.05 %.

### Number of panicles per plant

One QTL associated with panicles per plant was detected at both sites. This QTL was located near RM17308 on Chr.4 and contributed a small positive effect, increasing panicles per plant.

### Number of grains per panicle

One QTL located near RM184 on Chr.7 was associated with number of grains per panicle. This QTL conferred a negative effect, decreasing the number of grains per panicle.

### Grain length

Three QTLs associated with grain length were detected. QTLs near RM467 on Chr.10 and RM519 on Chr.12 contributed an increasing effect on grain length, while the QTL near RM15382 on Chr. 3 displayed a decreasing effect. The phenotypic variation explained by these three QTLs ranged from 6.07 to 15.3 %.

### Grain width

Four QTLs associated with grain width were detected at both sites. The phenotypic variation explained by these QTLs ranged from 6.08 to 19.0 %. Three QTLs, located near RM514, RM544, and RM128 on chromosomes 3, 8, and 1, respectively contributed a negative effect, while the QTL near RM3467 on Chr. 3 contributed a positive effect.

### 1000-grain weight

Two QTLs were detected as controlling grain weight; these were located near markers RM105 on Chr.9 and RM188 on Chr.5. Both had a decreasing effect on grain weight and the phenotypic variation explained by these QTLs ranged from 8.07 to 11.9 %.

### Length of flag leaf

Two QTLs were associated with flag leaf length. They were located near RM280 on Chr.4 and RM17954 on Chr.5. Both showed an increasing effect. The individual locus explained 4.50-18.6 % of phenotypic variation.

### Width of flag leaf

Two QTLs, located near RM206 on Chr.11 and RM128 on Chr.1, were associated with flag leaf width. Both showed a decreasing effect. The phenotypic variation explained by these QTLs ranged from 6.48 to 8.58 %.

### SNP detection for mapping of the heading date and seed shattering QTLs

**Fig. 5:**
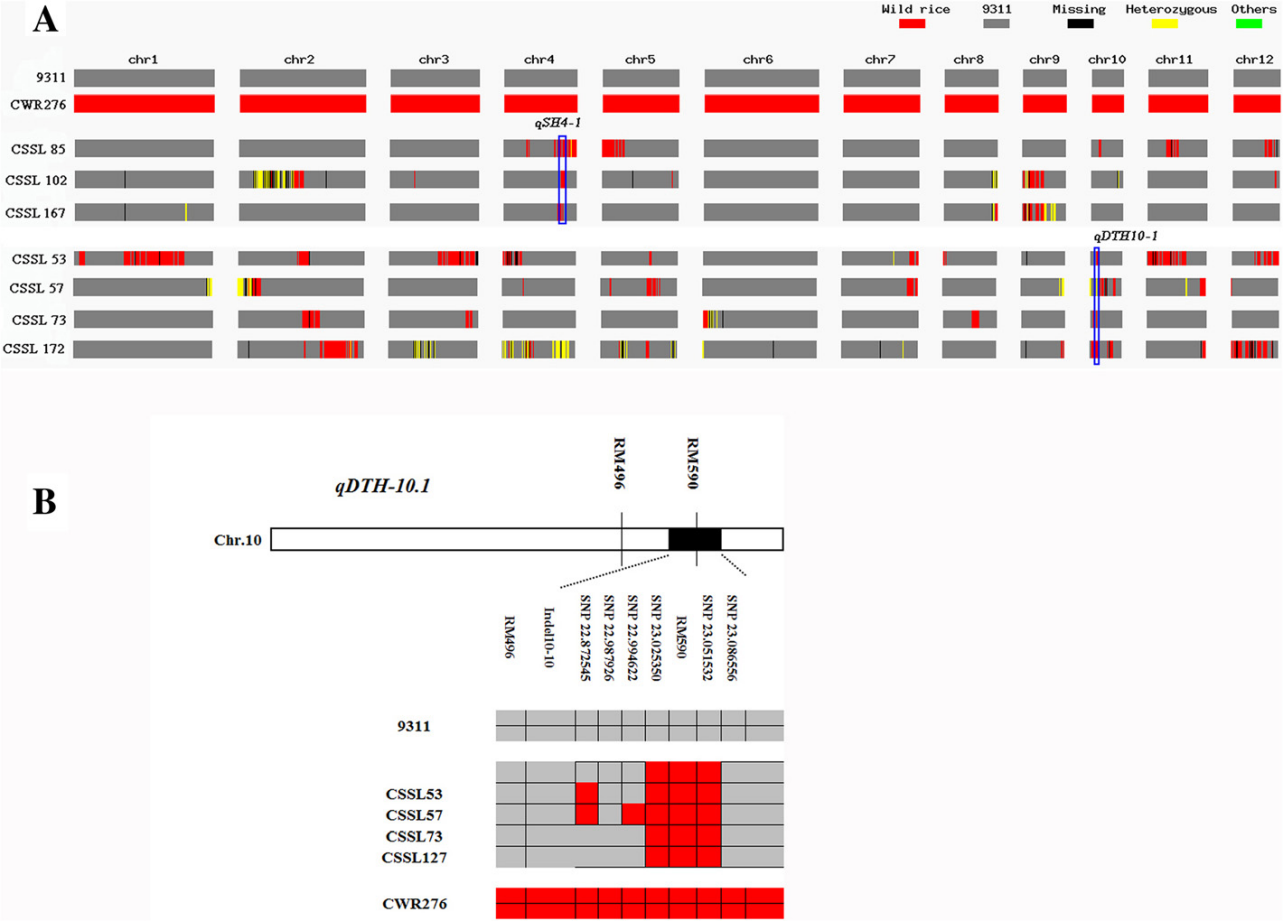
Fine mapping of QTLs for seed shattering and days to heading using a SNP array. (**a**) Genotyping of the two parents and seven CSSLs by SNP chip scanning. Blue rectangles represent the region where the wild rice genotype was found in all representative CSSLs. (**b**) Fine mapping of the days to heading QTL on Chr.10. Numbers following an SNP indicate the physical distance (Mb) of each SNP marker from the upper end of each chromosome. The SSR markers RM496, RM590 and marker InDel10-10 were located at position 22.5 Mb, 23.04 Mb and 22.7 Mb on Chr.10, respectively

To both confirm SSR marker genotyping and for fine mapping of QTLs associated with the heading date and seed shattering, seven CSSLs were selected for further SNP detection by whole-genome SNP chip scanning. SNP genotyping was performed using an Illumina SNP chip RiceSNP containing 9858 SNPs. Approximately 1500 SNPs were detected as polymorphic between the two parents (data not shown). CSSL85, 102, and 167 have significant seed shattering and were detected for seed shattering QTLs, while CSSL 53, 57, 73, and 172 have a significantly delayed heading phenotype, and thus were detected for heading date QTLs. One of the seed shattering QTLs, closest to marker RM349 on Chr.4, was identified in all three seed shattering CSSLs (Fig. 5a). Furthermore, one of the heading date QTLs, closest to marker RM590 on Chr.10, was identified in all four CSSLs with delayed heading. According to the positions of SNP markers near RM349, and information from a previous study [12], a *sh4* gene was found near RM349 on Chr.4 (Additional file 3: Figure S5). *qDTH10-1* was identified in a 78-kb region in Chr.10 according to the SNP markers and RM590 position (Fig. 5b); no gene has been reported in this region. Therefore, *qDTH10-1* is a new QTL associated with the heading date.

## Discussion

### Wild rice CSSL development and genome coverage

An excellent population of genetic materials is important for a comprehensive understanding of quantitative traits. CSSL have the potential to construct genome-wide genetic stocks, to identify QTLs for functional genomics research, and to discovering novel genes from newfound wild rice accessions. So far, CSSLs derived from wild speices of *Oryza* family including *O. meridionalis*, *O. glumepatula*, *O. rufipogon*, and *O. glaberrima* have been constructed [42–44]. Although the development of CSSLs for *O. rufipogon* has previously been reported [13, 38–40], diverse accessions should be included in such a genetic survey to retrieve genes lost through domestication or evolution. In this study, we developed a set of CSSLs derived from *O. rufipogon* in the genetic background of an elite cultivated rice. The donor parent CWR276 comes from the lowest latitudes area of China, and is considered to be the unique tropical *O. rufipogon* population in China. It has been characterized with representative morphological characteristics of Chinese common wild rice, including resistance or tolerance to multiple biotic and abiotic stresses. The primary objective of this study was to develop a wide population of genetic materials for rice breeding and genomic research.

The density of polymorphic markers throughout the entire genome plays a vitally important role in the development of CSSLs, the higher the marker density, the lower the number of donor chromosomal segments lost. Construction of CSSLs using MAS is well documented. Ebitani *et al*. constructed and genotyped a set of CSSLs using 129 RFLP markers [27]; Tian *et al.* used 126 SSR markers [13]; Takai *et al.* used 140 SSR and 5 EST markers [31]; Zhu *et al.* used 132 SSR markers [45]; Zhu *et al*. used 260 SSR and sequence tagged site markers [46], and Xu *et al*. used 254 markers, including SSR and InDel [29]. This study made use of 313 polymorphic markers fairly evenly distributed across the 12 rice chromosomes, representing a higher density than previously reported. Compared with re-sequencing-based high-throughput methods, the molecular marker-based genotyping approach is tedious and labor intensive. However, the CSSL population developed using high-density molecular markers provides starting material for advanced secondary population construction and QTL analysis. MAS was started at the BC_3_ generation, as some small chromosome segments were missed in advanced backcrosses the final coverage of the wild rice genome was about 85 % in the 198 CSSLs population. Forty percent of the substituted segments were smaller than 5 cM, and about 36 % of CSSLs had only one substituted segment that could be considered as NILs. Data demonstrated these CSSLs were better than the aforementioned wild rice CSSLs on both lengths of introgressed segments and amount of NILs. The uncovered regions in this set of CSSLs might have occurred because MAS was not performed until the BC_3_ generation, some biological factors such as gametophyte, hybrid sterility and heading-date genes might also be considered the cause. Tracing back to an earlier generation, such as BC_2_, to fill the gaps should make it possible for this CSSL set to cover the entire wild rice genome.

### CSSLs provided a platform for both rice breeding and QTL mapping

Although molecular tools and sequencing technique have rapidly been developed, phenotyping remains the most crucial and challenging factor in genetic analysis of complex traits. The two parents used in this study have a strong potential for heterosis. Thus, the CSSLs showed a very large variation in all agronomic traits. Although wild rice is generally inferior to cultivated rice in terms of yield traits, previous reports demonstrated there are many high-yielding QTLs in low-yielding wild rice [13, 47, 48]. Transgressive segregation of all yield traits was observed in these CSSLs, especially for the number of panicles per plant and grain length, which on average exceeded that of recurrent parent 9311 at the two sites (Table 3). Cultivar 9311 has been planted on a large scale in China as it is an elite variety. Some CSSLs in this stduy had a similar genetic background as 9311, but their comprehensive characteristics were better than 9311 under different environmental conditions. Therefore, they could be directly used to develop new varieties, or as a parent to produce new superior hybrids. These CSSLs could provide a useful material population for cultivated rice breeding.

CSSLs can ben used to facilitate detecting and fine mapping of QTLs by eliminating genetic background noise, this would simplify the data analysis process and increase the accuracy of results. In this study, CSSLs were used for QTLs mapping of 10 agronomic traits, and 25 QTLs were identified as present at both Nanjing and Sanya sites. Some QTLs were either reported or contained previously reported genes (Additional file 2: Table S3). SNP chip scanning should prove an efficient tool for QTL confirmation. The SNP chip used in this study was selected from two parents *indica* and *japonica* [49], thus only about 15 % of SNP sites were detected as polymorphic between 9311 and CWR276. Two traits, days to heading and seed shattering, which are considered to be the most important traits involved in rice domestication, were selected for further fine mapping using whole-genome SNP chip scanning. The QTL *qSH4-1* associated with seed shattering was identified in Chr. 4 and a previously reported gene *sh4* has been found in this location [12]. The location of another QTL, *qDTH10-1*, associated with the heading date was narrowed down to a 78-kb region on Chr.10 (Fig. 5b); no previously reported gene is found in this region. This QTL could be used for new heading date gene searching. Generically, heading date often influences many other agronomic traits, however, the *qDTH10-1* did not show pleiotropic effects to other agronomic traits (Fig. 1 and Additional file 2: Table S3), this QTL might be useful to alter heading date without influences other agronomic traits in rice breeding. The results suggest the CSSLs in this study will prove an efficient population for QTL identification, and the use of different wild rice accessions would lead to discovery of novel genes.

Plant breeding requires the combination of art and science to improve the genetic basis of new crop varieties so as to incorporate better agronomic and yield traits. Systems of plant breeding using molecular MAS to combine phenotype and genotype have entered a new era. The development of wild rice CSSLs has provided a broad platform for both wild rice genomic research and QTL mapping. Novel genes found in wild rice using these CSSLs could provide a new genetic resource for breakthroughs in rice breeding.

## Conclusion

We successfully developed a wide population containing 198 CSSLs from wild rice in the genetic background of elite *indica* cultivar 9311. The whole CSSL population covered approximately 85 % of the wild rice genome with an average substituted segment length of 6 cM. Each CSSL contained an average of two introgressed segments. Abundant QTLs associated with agronomic traits were identified based on an evaluation of phenotypic variation and genotyping of 313 SSR markers. Combined with a SNP chip assay, a novel small QTL associated with the heading date was fine mapped in the selected CSSL. The CSSLs described in this study could prove a powerful tool for large-scale gene discovery and provide an important germplasm resource for rice breeding.

## Methods

### Plant materials

Development of CSSLs made use of the commercial elite restorer *indica* cultivar 9311 as the recipient. 9311 is characterized by its high yield, eating quality, and resistance to multiple diseases. Chinese common wild rice accession CWR276 (*O. rufipogon*) was collected from Sanya, Hainan Province, and used as the donor parent. The ratoon was collected from its original habitat and conserved in our wild rice germplasm garden. The photo of two parents was showed in S-Figure1.

### Construction of CSSLs

The F_1_ plant derived from a cross between 9311 and CWR276 was backcrossed to 9311 to produce 176 BC_1_F_1_ plants. BC_1_ plants were then backcrossed to 9311 two times, without MAS, to produce a BC_3_F_1_ generation. Whole-genome genotyping was performed in the BC_3_F_1_ generation using SSR markers distributed across the 12 rice chromosomes. One thousand BC_3_F_1_ plants were surveyed by 230 SSR markers, and 236 plants selected for backcrossing with 9311. According to SSR genotypes, the BC_4_ generation was selfed or backcrossed consecutively to generate BC_4_F_5_, or BC_5_F_4_, BC_6_F_4_, and BC_7_F_4_. Finally, 198 lines were used to construct the CSSLs.

### DNA isolation and PCR

Genomic DNA was extracted from freshly frozen leaves of individuals using the CTAB method described by Rogers and Bendich [50]. Extracted DNA was stored in ddH_2_O at −20 °C.

SSR marker primers were selected from dense rice microsatellite maps, and synthesized in accordance with sequences published by Ware *et al.* [41] or Temnykh *et al.* [51]. Some markers were designed according to information available from Gramene. DNA amplification was performed using PCR with the following conditions: 95 °C for 5 min; 33 cycles of 94 °C for 30 s, 55 °C for 30 s, and 72 °C for 30 s; and a final cycle of 72 °C for 10 min. Reactions were carried out in 96-well PCR plates in 25-μL volumes containing 1 μmol/L of each primer, 200 μmol/L of dNTPs, 5 ng of DNA template, 2 mmol/L MgCl_2_, 2.5 μL 10× buffer, and 1 U of Taq polymerase (Dong-Sheng Limited, Beijing,China). PCR products were separated on 8 % polyacrylamide denaturing gels, and bands visualized using the silver-staining protocol described by Panaud *et al*. [52]. Some amplification products were analyzed on 3.5 % agarose gels stained with ethidium bromide and photographed using a UVP system.

### Determination of length of substituted segments in CSSLs

A genetic linkage map was built to estimate marker distances with reference to Temnykh *et al.* and Ware *et al.* [41, 51]. The lengths of substituted chromosome segments in CSSLs were determined based on graphical genotypes [29, 53]. Construction of graphical genotypes and calculation of percentage of the total genome in each CSSL line were performed using GGT software [54]. A chromosome segment flanked by two markers of donor type (DD) was considered 100 % donor type; a chromosome segment flanked by two markers of recipient type (RR) was considered 0 % donor type; and a chromosome segment flanked by one marker of donor type and one marker of recipient type (DR) was considered 50 % donor type. The length of DD plus the length of two half DRs were considered the estimated length of a substituted chromosome segment.

### Measurement of agronomic traits

The phenotypic evaluation of 198 CSSLs was performed under natural conditions at the experimental stations of the Chinese Academy of Agricultural Science in summer (Nanjing, China; N32°03′, E118°47′) and winter (Sanya, China; N18°15′, E109°30′) 2014. The field experiment was designed in randomized plots with two replications. For each CSSL and the parents, 60 plants were planted in five rows, with 20 cm between plants within each row, and 30 cm between rows. Fifteen plants in the center of each plot were selected for the collection of data. Days to heading was deemed the number of days from sowing to heading of plants. Seed shattering was evaluated using the method described by Han and Wei [55]. Plant height, number of panicles per plant, and length and width of the flag leaf were the mean value of 20 randomly selected individuals. Number of grains per panicle, 1000-grain weight, and grain length and width were detected by an automatic seed investigation machine (Wanshen, Shenzhen, China). Differences between CSSLs and 9311 were determined using a *t-*test.

### CSSL-based QTL mapping and SNP assay

The association between phenotype and marker genotype was investigated by single-point analysis using Map Manager QTXb17 [56] and SPSS 13.0 (SPSS Inc., Chicago, IL, USA). The statistical threshold for single-point analysis was *P* < 0.01. Genotyping of the seven selected CSSLs and the two parents was performed using an Illumina SNP chip RiceSNP containing 9858 SNPs at the Shenzhen Academy of Crop Molecular Breeding, China. The mean distance between adjacent SNP markers was 54.5 kb. Chromosomal positions of SNPs were determined according to the 9311 reference genome.

## Additional files

Additional file 1: Table S2. Correlation coefficients among the ten agronomic traits. (DOCX 16 kb) Additional file 2 Table S3. QTLs for ten agronomic traits detected in 198 CSSLs at Nanjing and Sanya sites. Table S1: Sequence information of SSR primers used in this study. Figure S1: Photographs of *Oryza sativa spp. indica* cultivar 9311 and wild rice CWR276. Figure S2: Nucleotide (A) and amino acid (B) alignments of the sh4 gene between wild rice and 9311. Wild rice alleles were cloned from CSSL85, 102, 167, and the donor parent BC276. The red rectangle indicates a substitution (K in the protein of the wild rice allele). (DOCX 36 kb)

## Abbreviations

cM: centimorgan
CSSL: chromosome segment substitution line
IL: introgression line
MAS: marker-assisted selection
NIL: near isogenic line
QTL: quantitative trait locus
SNP: single nucleotide polymorphism
SSR: simple sequence repeat
