# Supplementary material for: Development and characterization of chromosome segment substitution lines derived from Oryza rufipogon in the genetic background of O. sativa spp. indica cultivar 9311
Source: BMC Genomics. 2016 Aug 9;17:580. doi: 10.1186/s12864-016-2987-5 (PMC4979106; doi:10.1186/s12864-016-2987-5)
Supplement: Additional file 1: Table S2. — Correlation coefficients among the ten agronomic traits. (DOCX 16 kb) [file 12864_2016_2987_MOESM1_ESM.docx]

S-Table 2 Correlation coefficients among the ten agronomic traits.

|  | 1000-grain weight (g) | Grain length (mm) | Grain width (mm) | Number of grains per panicle | Day to heading (days) | Plant height(cm) | Length of flag leaf(cm) | Width of flag leaf(cm) | Number of panicles per plant | Seeds shattering |
| --- | --- | --- | --- | --- | --- | --- | --- | --- | --- | --- |
| 1000-grain weight (g) | 1 |  |  |  |  |  |  |  |  |  |
| Grain length(mm) | 0.650^**^ | 1 |  |  |  |  |  |  |  |  |
| Grain width (mm) | 0.414^**^ | -0.011 | 1 |  |  |  |  |  |  |  |
| Number of grains per panicle | -0.041 | -0.074 | -0.066 | 1 |  |  |  |  |  |  |
| Day to heading (days) | 0.147^*^ | 0.169^*^ | 0.244^**^ | -0.125 | 1 |  |  |  |  |  |
| Plant height(cm) | -0.141 | 0.034 | -0.339^**^ | 0.327^**^ | -0.169^*^ | 1 |  |  |  |  |
| Length of flag leaf(cm) | -0.221^**^ | -0.066 | -0.240^**^ | 0.313^**^ | 0.055 | 0.706^**^ | 1 |  |  |  |
| Width of flag leaf(cm) | 0.217^**^ | 0.114 | 0.285^**^ | 0.142 | 0.302^**^ | -0.346^**^ | -0.050 | 1 |  |  |
| Number of panicles per plant | -0.200^**^ | -0.066 | -0.074 | -0.353^**^ | 0.276^**^ | -0.287^**^ | -0.243^**^ | -0.071 | 1 |  |
| Seeds shattering | -0.161^*^ | -0.069 | -0.075 | -0.241^**^ | 0.243^**^ | 0.018 | 0.121 | 0.044 | 0.236^**^ | 1 |
| Asterisks indicate significant correlation coefficients: *, p ≤0.05; **, p ≤ 0.01. | | | | | | | | | | |
