# Supplementary material for: Development and characterization of chromosome segment substitution lines derived from Oryza rufipogon in the genetic background of O. sativa spp. indica cultivar 9311
Source: BMC Genomics. 2016 Aug 9;17:580. doi: 10.1186/s12864-016-2987-5 (PMC4979106; doi:10.1186/s12864-016-2987-5)
Supplement: Additional file 2 Table S3. — QTLs for ten agronomic traits detected in 198 CSSLs at Nanjing and Sanya sites. Table S1: Sequence information of SSR primers used in this study. Figure S1: Photographs of Oryza sativa spp. indica cultivar 9311 and wild rice CWR276. Figure S2: Nucleotide (A) and amino acid (B) alignments of the sh4 gene between wild rice and 9311. Wild rice alleles were cloned from CSSL85, 102, 167, and the donor parent BC276. The red rectangle indicates a substitution (K in the protein of the wild rice allele). (DOCX 36 kb) [file 12864_2016_2987_MOESM2_ESM.docx]

S-Table 3 **QTLs for ten agronomic traits detected in 198 CSSLs at Nanjing and Sanya sites.**

| Trait | QTL | Marker | Chr. | Marker Position (bp) | Envionment | LOD | PVE (%) | Add | QTL reported (QTL Accession) | Ref. |
| --- | --- | --- | --- | --- | --- | --- | --- | --- | --- | --- |
| Days to heading | *qDTH2-1* | RM485 | 2 | 934,340-934,590 | Nanjing | 2.97 | 17.2 | 20.6 | *None* |  |
|  |  |  |  |  | Sanya | 2.67 | 10.5 | 14.5 |  |  |
|  | *qDTH2-2* | RM535 | 2 | 35,778,242-35,778,527 | Nanjing | 4.65 | 7.89 | 18.9 | *AQA029* | [1](http://archive.gramene.org/db/literature/pub_search?ref_id=6917) |
|  |  |  |  |  | Sanya | 5.94 | 5.38 | 12.5 |  |  |
|  | *qDTH10-1* | RM590 | 10 | 23,043,156-23,043,293 | Nanjing | 2.86 | 15.3 | 21.2 | *AQED025 etc* | 2 |
|  |  |  |  |  | Sanya | 2.12 | 10.2 | 5.60 |  |  |
|  | *qDTH12-1* | RM19 | 12 | 2,433,226 -2,433,554 | Nanjing | 4.00 | 5.98 | 3.04 | *AQFW164 etc* | 3 |
|  |  |  |  |  | Sanya | 2.38 | 2.66 | 1.67 |  |  |
|  | *qDTH10-2* | RM216 | 10 | 5,352,766-5,352,898 | Nanjing | 5.22 | 9.86 | -4.35 | *AQAM031 etc* | 4 |
|  | *qDTH12-2* | RM28449 | 12 | 22,622,621-22,622,966 | Nanjing | 3.67 | 8.73 | -2.05 | *None* |  |
|  | *qDTH6-1* | RM20438 | 6 | 25,570,271-25,570,397 | Sanya | 2.95 | 6.53 | -1.50 | *None* |  |
| Seed shattering | *qSH2-1* | RM7288 | 2 | 9,033,547-9,033,882 | Nanjing | 2.51 | 5.26 | 1.09 | *None* |  |
|  |  |  |  |  | Sanya | 2.99 | 4.98 | 1.21 |  |  |
|  | *qSH4-1* | RM349 | 4 | 32,499,412-32,499,619 | Nanjing | 3.48 | 8.54 | 1.66 | *AQDU001 etc* | 5 |
|  |  |  |  |  | Sanya | 5.22 | 9.65 | 2.73 |  |  |
|  | *qSH5-1* | RM289 | 5 | 7,807,745-7,807,830 | Nanjing | 2.72 | 5.03 | -0.75 | *AQA031 etc* | 1 |
|  |  |  |  |  | Sanya | 2.65 | 3.59 | -0.72 |  |  |
|  | *qSH4-2* | RM273 | 4 | 30,772,388-30,772,538 | Nanjing | 2.88 | 6.93 | 1.30 | *AQAM010 etc* | 4 |
|  |  |  |  |  | Sanya | 2.57 | 7.23 | 1.56 |  |  |
| Plant height | *qPH1-1* | RM128 | 1 | 28,339,373-30,870,559 | Nanjing | 3.19 | 7.00 | 15.8 | *AQA018 etc.* | 1 |
|  |  |  |  |  | Sanya | 3.07 | 6.85 | 12.6 |  |  |
|  | *qPH1-2* | RM473 | 1 | 28,135,218-28,135,119 | Nanjing | 4.45 | 9.04 | 25.3 | *AQA018 etc.* | 1 |
|  |  |  |  |  | Sanya | 3.55 | 7.63 | 20.3 |  |  |
|  | *qPH2-1* | RM6318 | 2 | 24,420,594-24,420,792 | Nanjing | 2.56 | 5.20 | -17.5 | *None* |  |
|  |  |  |  |  | Sanya | 2.89 | 4.32 | -16.8 |  |  |
|  | *qPH4-1* | RM280 | 4 | 34,989,558-34,989,727 | Nanjing | 3.67 | 8.42 | 34.4 | *AQEI057 etc.* | 6 |
|  |  |  |  |  | Sanya | 3.57 | 6.76 | 20.3 |  |  |
|  | *qPH6-1* | RM412 | 6 | 30,327,854-30,328,051 | Sanya | 3.79 | 7.67 | 42.2 | *AQB008* | 7 |
|  | *qPH4-2* | RM127 | 4 | 34,529,722-34,529,916 | Nanjing | 3.06 | 6.15 | 14.5 | *AQFF038* | 8 |
|  | *qPH10-1* | RM216 | 10 | 5,352,766-5,352,898 | Nanjing | 4.45 | 9.61 | 37.8 | *AQAM031 etc* | 4 |
|  | *qPH3-1* | RM85 | 3 | 27,411,671-33,386,423 | Sanya | 10.9 | 22.0 | 19.9 | *AQCD002 etc.* | 9 |
|  | *qPH7-1* | RM481 | 7 | 28,76,165-28,76,314 | Sanya | 4.38 | 8.09 | -19.6 | *AQFB005 etc.* | 10 |
| Panicles per plant | *qPPP4-1* | RM17308 | 4 | 27,282,047-27,282,196 | Nanjing | 2.75 | 5.65 | 0.88 | *None* |  |
|  |  |  |  |  | Sanya | 2.59 | 5.02 | 0.76 |  |  |
|  | *qPPP7-1* | RM481 | 7 | 28,76,165-28,76,314 | Nanjing | 3.83 | 9.11 | 1.42 | *AQFB005 etc.* | 10 |
|  | *qPPP7-2* | RM180 | 7 | 5,735,196-5,735,385 | Sanya | 3.32 | 7.14 | -0.75 | *AQAM041 etc.* | 4 |
| Number of grains per panicle | *qGPP7-1* | RM184 | 7 | 16,358,895-16,359,111 | Nanjing | 2.58 | 3.53 | -50.2 | *None* |  |
|  |  |  |  |  | Sanya | 3.01 | 3.25 | -48.9 |  |  |
|  | *qGPP7-2* | RM125 | 7 |  | Nanjing | 3.18 | 7.62 | -31.1 | *AQA024 etc.* | 1 |
|  | *qGPP3-1* | RM571 | 3 | 25,128,239-30,912,974 | Nanjing | 3.14 | 7.53 | -21.7 | *AQED044 etc.* | 2 |
| Grain length | *qGL3-1* | RM15382 | 3 | 22,166,710-22,166,808 | Nanjing | 6.26 | 14.9 | -0.45 | *None* |  |
|  |  |  |  |  | Sanya | 5.46 | 12.5 | 0.76 |  |  |
|  | *qGL10-1* | RM467 | 10 | 13,488,471-13,488,769 | Nanjing | 3.10 | 6.07 | 0.11 | *AQGA040 etc.* | 11 |
|  |  |  |  |  | Sanya | 3.07 | 5.88 | 1.23 |  |  |
|  | *qGL12-1* | RM519 | 12 | 19,903,791-19,903,912 | Nanjing | 6.55 | 15.3 | 0.26 | *AQFB013 etc.* | 10 |
|  |  |  |  |  | Sanya | 6.05 | 13.2 | 1.35 |  |  |
|  | *qGL8-1* | RM447 | 8 | 26,546,992-26,547,102 | Nanjing | 3.09 | 5.75 | -0.15 | *AQA010 etc.* | 1 |
|  | *qGL4-1* | RM537 | 4 | 185,131-185,366 | Nanjing | 6.92 | 15.8 | -0.75 | *AQGH012 etc.* | 12 |
| Grain width | *qGW3-2* | RM514 | 3 | 35,281,232-35,281,722 | Nanjing | 5.22 | 11.2 | -0.15 | *AQHF020 etc.* | 13 |
|  |  |  |  |  | Sanya | 5.05 | 9.23 | -0.14 |  |  |
|  | *qGW8-1* | RM544 | 8 | 5,108,207-5,108,454 | Nanjing | 2.99 | 6.07 | -0.06 | *AQA009 etc.* | 1 |
|  |  |  | 8 |  | Sanya | 4.06 | 7.86 | -0.13 |  |  |
|  | *qGW1-1* | RM128 | 1 | 28,339,373-30,870,559 | Nanjing | 5.78 | 11.3 | -0.10 | *AQA018 etc.* | 1 |
|  |  |  | 1 |  | Sanya | 2.99 | 6.58 | -1.33 |  |  |
|  | *qGW3-1* | RM3467 | 3 | 6,003,496-6,003,618 | Nanjing | 8.14 | 19.0 | 0.10 | *None* |  |
|  |  |  | 3 |  | Sanya | 6.93 | 15.8 | 0.21 |  |  |
|  | *qGW3-2* | RM85 | 3 | 32,674,852-32,675,116 | Nanjing | 2.79 | 6.72 | -0.06 | *AQJ003* | 13 |
| 1000-grain weight | *qTGW9-1* | RM105 | 9 | 11,807,446-15,662,838 | Nanjing | 3.52 | 8.06 | -1.23 | *AQA015* | 1 |
|  |  |  |  |  | Sanya | 4.76 | 10.9 | -1.00 |  |  |
|  | *qTGW5-1* | RM188 | 5 | 22,671,210-22,671,419 | Nanjing | 6.05 | 11.8 | -1.50 | *AQA032 etc.* | 1 |
|  |  |  |  |  | Sanya | 5.31 | 12.3 | -1.98 |  |  |
|  | *qTGW4-1* | RM537 | 4 | 185,131-185,366 | Nanjing | 6.23 | 14.3 | -3.46 | *AQGH012* | 12 |
| Length of flag leaf | *qLFL4-1* | RM280 | 4 | 34,989,558-34,989,727 | Nanjing | 9.08 | 18.6 | 10.5 | *AQEI057* | 6 |
|  |  |  |  |  | Sanya | 4.94 | 10.2 | 5.37 |  |  |
|  | *qLFL5-1* | RM17954 | 5 | 3,651,365-3,651,562 | Nanjing | 2.53 | 4.49 | 2.62 | *None* |  |
|  |  |  |  |  | Sanya | 2.55 | 2.39 | 1.63 |  |  |
|  | *qLFL4-2* | RM537 | 4 | 185,131-185,366 | Sanya | 5.29 | 10.1 | -5.16 | *AQGH012* | 12 |
|  | *qLFL4-3* | RM127 | 4 | 34,529,722-34,529,916 | Nanjing | 3.16 | 7.57 | 2.59 | *AQFF038* | 8 |
| Width of flag leaf | *qWFL11-1* | RM206 | 11 | 22,014,679-22,014,851 | Nanjing | 3.87 | 8.58 | -0.08 | *AQAP050* | 15 |
|  |  |  |  |  | Sanya | 3.01 | 6.32 | -1.56 |  |  |
|  | *qWFL1-1* | RM128 | 1 | 28,339,373-30,870,559 | Nanjing | 3.52 | 6.47 | -0.10 | *AQA018 etc.* | 1 |
|  |  |  |  |  | Sanya | 2.55 | 3.25 | -2.18 |  |  |

LOD: LOD score calculated from single marker analysis.

PVE(%): Phenotypic variation explained by the marker.

Add: Estimated additive effect of the marker.

**References:**

1. Hemamalini GS, Shashidhar HE, Hittalmani S: **Molecular marker assisted tagging of morphological and physiological traits under two contrasting moisture regimes at peak vegetative stage in rice (Oryza sativa L.)**. *Euphytica* 2000, 112 (1):69-78.
2. Septiningsih EM, Prasetiyono J, Lubis E, Tai T H, Tjubaryat T, Moeljopawiro S, McCouch SR: **Identification of quantitative trait loci for yield and yield components in an advanced backcross population derived from the Oryza sativa variety IR64 and the wild relative O. rufipogon.** *Theor Appl Genet* 2003, 107(8):1419-1432.
3. Mei HW, Luo LJ, Ying CS, Wang YP, Yu XQ, Guo LB, Paterson AH, Li ZK: **Gene actions of QTLs affecting several agronomic traits resolved in a recombinant inbred rice population and two testcross populations.** *Theor Appl Genet* 2003, 107(1):89-101.
4. Hu FY, Tao DY, Sacks E, Fu BY, Xu P, Li J, Yang Y, McNally K, Khush GS, Paterson AH, Li ZK: **Convergent evolution of perenniality in rice and sorghum.** *Proc Natl Acad Sci USA* 2003, 100(7):4050-4054.
5. Kashiwagi T, Ishimaru K: **Identification and functional analysis of a locus for improvement of lodging resistance in rice**. *Plant physiol* 2004, 134(2):676-683.
6. Jiang GH, He YQ, Xu CG, Li XH, Zhang Q: **The genetic basis of stay-green in rice analyzed in a population of doubled haploid lines derived from an indica by japonica cross.** *Theor Appl Genet* 2004, 108(4):688-698.
7. Bao JS, Wu YR, Hu B, Wu P, Cui HR, Shu QY: **QTL for rice grain quality based on a DH population derived from parents with similar apparent amylose content.** *Euphytica* 2002, 128(3):317-324.
8. Lanceras JC, Pantuwan G, Jongdee B, Toojinda T: **Quantitative trait Loci associated with drought tolerance at reproductive stage in rice.** *Plant physiol* 2004, 135(1):384-399.
9. Yan CJ, Xu CW, Yi CD, Liang GH, Zhu LH, Gu MH: **Genetic analysis of gelatinization temperature in rice via microsatellite (SSR) markers.** *Acta genetica Sinica* 2001, 28(11):1006-1011.
10. Gu XY, Kianian SF, Foley ME: **Multiple loci and epistases control genetic variation for seed dormancy in weedy rice (Oryza sativa).** *Genetics* 2004, 166(3):1503-1516.
11. Fan CC, Yu XQ, Xing YZ, Xu CG, Luo LJ, Zhang Q: **The main effects, epistatic effects and environmental interactions of QTLs on the cooking and eating quality of rice in a doubled-haploid line population.** *Theor Appl Genet* 2005, 110(8):1445-1452.
12. Tian F, Li DJ, Fu Q, Zhu ZF, Fu YC, Wang XK, Sun CQ: **Construction of introgression lines carrying wild rice (Oryza rufipogon Griff.) segments in cultivated rice (Oryza sativa L.) background and characterization of introgressed segments associated with yield-related traits.** *Theor Appl Genet,* 2006, **112**(3): 570-580.
13. Yue B, Xue WY, Luo LJ, Xing YZ: **QTL analysis for flag leaf characteristics and their relationships with yield and yield traits in rice**. *Acta Genetica Sinica* 2006, 33(9):824-832.
14. Lafitte HR, Courtois B, Arraudeau M: **Genetic improvement of rice in aerobic systems: Progress from yield to genes.** *Field Crops Res* 2002, 75(2):171-190.
15. Xu XF, Mei HW, Luo LJ, Cheng XN, Li ZK: **RFLP-facilitated investigation of the quantitative resistance of rice to brown planthopper ( *Nilaparvata lugens*).** *Theor Appl Genet* 2002, 104(2):248-253.
